# Reconfigurable Radiation Pattern of Planar Antenna Using Metamaterial for 5G Applications

**DOI:** 10.3390/ma13030582

**Published:** 2020-01-26

**Authors:** Bashar Ali Esmail, Huda A Majid, Zuhairiah Zainal Abidin, Samsul Haimi Dahlan, Mohamed Himdi, Raimi Dewan, Mohamad Kamal A Rahim, Najib Al-Fadhali

**Affiliations:** 1 Center for Applied Electromagnetic, Universiti Tun Hussein Onn Malaysia, Batu Pahat 86400, Malaysiazuhairia@uthm.edu.my (Z.Z.A.); samsulh@uthm.edu.my (S.H.D.); eng.najeebfadhali@gmail.com (N.A.-F.); 2 Institute of Electronics and Telecommunication of Rennes, University of Rennes 1, 35000 Rennes, France; mohamed.himdi@univ-rennes1.fr (M.H.); raimi-bin.dewan@univ-rennes1.fr (R.D.); 3 School of Electrical Engineering, Faculty of Engineering, Universiti Teknologi Malaysia, 81310 Skudai, Johor, Malaysia; mdkamal@utm.my

**Keywords:** beam deflection, 5G, millimeter-wave (MMW), reconfigurable metamaterial

## Abstract

In this research, a reconfigurable metamaterial (MM) structure was designed using a millimeter-wave (MMW) band with two configurations that exhibit different refractive indices. These two MM configurations are used to guide the antenna’s main beam in the desired direction in the 5th generation (5G) band of 28 GHz. The different refractive indices of the two MM configurations created phase change for the electromagnetic (EM) wave of the antenna, which deflected the main beam. A contiguous squares resonator (CSR) is proposed as an MM structure to operate at MMW band. The CSR is reconfigured using three switches to achieve two MM configurations with different refractive indices. The simulation results of the proposed antenna loaded by MM unit cells demonstrate that the radiation beam is deflected by angles of +30° and −27° in the E-plane, depending on the arrangement of the two MM configurations on the antenna substrate. Furthermore, these deflections are accompanied by gain enhancements of 1.9 dB (26.7%) and 1.5 dB (22.4%) for the positive and negative deflections, respectively. The reflection coefficients of the MM antenna are kept below −10 dB for both deflection angles at 28 GHz. The MM antennas are manufactured and measured to validate the simulated results.

## 1. Introduction

The rapid increase in the number of wireless service users has created serious challenges for telecommunications industries regarding bandwidth scarcity in current networks. Therefore, service providers have moved toward fifth-generation (5G) networks to meet these requirements. 5G networks provide data rates of up to 1000 times higher and bandwidth 10 times greater than current communication networks [1]. The well-known spectrum candidate for delivering 5G is millimeter-wave (MMW), which includes bands such as 28 and 60 GHz. Although these bands provide multigigabits-per-second data rates and high bandwidth, they experience very high path loss based on Friis’s formula, which limits the range of communications to short-range distances when compared to sub-6 GHz frequencies [2]. To overcome this problem, the high-gain directional antenna should be incorporated into both communication system terminals to overcome the greater path loss and enhance link quality. Deflecting an antenna’s radiation pattern in a predefined direction is very important for enhancing the performance of communication systems in terms of the quality of service, system security, avoiding interference, and economizing power [3]. In the literature, the mechanical and electronic approaches have been proposed as conventional methods to perform beam tilting at the base station and mobile station. Despite the drawbacks of bulky structure and low switching speed of the mechanical method, it provides a large scan angle in comparison with other methods [4]. On the other hand, the electronic method provides high switching speed and small physical structure. However, it suffers from inherent high loss due to the active components used [5,6]. The phased array antenna and butler matrix networks are also used to guide the radiation pattern in the required direction. However, these approaches suffer from bulky, high cost, and complex transceiver system [7,8]. Moreover, the drop in the gain is a common issue in most of the conventional beam deflection methods.

On the other hand, various additional materials had been proposed to reconfigure the MM structures, such as graphene and liquid crystal. This method provides low-cost tunability and low loss in comparison with other methods. In [9], the authors propose an MM with a single layer of graphene placed on its surface. The proposed MM exhibits exceptional sensitivity to the presence of the graphene layer. The graphene dramatically alters the transmission spectrum of the MM structure, thereby controlling the loss of such materials. The MM that containing a multilayer of graphene material had been implemented at far- and mid-infrared spectrums [10]. This implementation shows promising features such as tuning of the MM. The tunable and controllable transition from hyperbolic to elliptic dispersion was implemented using electrostatic biasing. In [11], the reconfigurable MM had been implemented by including a liquid crystal layer. By reorienting the liquid crystal layer between the split square resonator (SSR), the bandwidth and unique properties of MM, such as the refractive index, can be controlled. Also in [12], the authors proposed that the MM structure that comprises liquid crystal for achieving the reconfiguration. The liquid crystal was placed into silicon layers. By applying the AC bias voltage between these layers, the permittivity and the loss of MM can be controlled.

Recently, MM structures are integrated with planar antennas for beam deflection applications. Many noteworthy properties are realized in these artificial materials, such as negative refractive index and inverse Doppler shift, due to negative permittivity and permeability [13]. MMs have been extensively explored because of their versatility as perfect absorbers [14], superlens [15], cloaking devices [16], and polarization converters [17]. However, many reports have found that MM possess high insertion loss and strict bandwidth, which are the main drawbacks affecting the domain of their applications [18]. The integration of MM with a planar antenna can enhance the antenna’s performance in terms of gain, bandwidth, and efficiency [19,20]. Beam deflection is an interesting application of these human-made materials. For this, a conventional split-ring resonator (SRR) is used to tilt the patch antenna’s beam by +15° in the C-band with a physical size of 1.35$\;\lambda_{0\;}$× 1.26$\;\lambda_{0}\;$× 0.4$\;\lambda_{0}$ [21]. However, the main beam is tilted by +15° toward one direction only. Further, the gain declines by 1.5 GHz when the beam is tilted. Also, a fixed deflection angle of +17° can be achieved using a bow-tie antenna loaded by an H-shape MM with dimensions of 1$\lambda_{0}\;$× 1.5$\;\lambda_{0\;}$× 0.04$\;\lambda_{0}$ in the C-band [22]. Although there is gain enhancement through the deflection process, the tilting angle is limited to 17° in a positive direction only. In [23], the authors combined SRR and H-shape in one-unit cell and used the array of MM unit cells to tilt the radiation beam of a horn antenna by an angle of +10°. This structure had a large physical size, i.e., 3.6$\;\lambda_{0}\;$× 5.6$\;\lambda_{0\;}$× 0.2$\;\lambda_{0}$. Further, the gain decreases when a small deflection angle is achieved. The radiation pattern of a dipole antenna was deflected in [24] using high-refractive index metamaterial (HRIM). Fixed beam deflection in one direction by an angle of +30° in the V-band has been achieved. The authors in [25] included a periodic J-shape MM into a leaky-wave antenna to deflect the radiation beam in both directions at angles of ±15°. Also, in [26], the radiation pattern of the proposed antenna was deflected at angles of 25° and −24° using an array of the adjacent square-shaped resonator (ASSR).

In this paper, an MM structure with reconfigurable property has been integrated with a printed dipole antenna to deflect the radiation beam with gain enhancement in positive and negative directions (+y direction or −y direction). The radiation beam is deflected to a high refractive index section (ON configuration). The proposed antenna operates at 28 GHz, which has an acceptable path loss and bandwidth compared to higher MMW frequencies. Two MM configurations are arranged in the vicinity of the dipole radiating elements to guide the main beam in desired directions at angles of +30° and−27°. These deflections were achieved with gain enhancements of 26.7% and 22.4% for positive and negative deflection angles, respectively.

## 2. Design and Characterization of the Proposed MM Structure 

**Figure 1:**
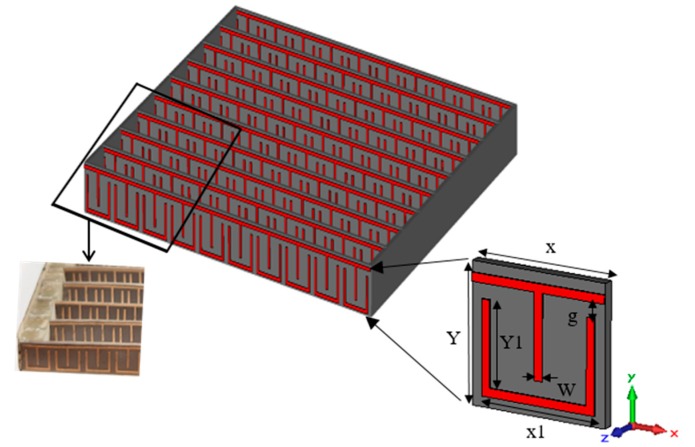
Schematic view of the contiguous squares resonator (CSR) periodic structure with the geometry of the single metamaterial (MM) unit cell and fabricated prototype.

The configuration of the contiguous squares resonator (CSR) periodic structure with the geometry of the single MM unit cell and fabricated prototype are displayed in Figure 1. The capacitance and inductance effects have been induced by the gaps and square loops of the structure, which can be adjusted through the simulation to control the resonance characteristics of the structure. The Rogers RT5880 (relative permittivity = 2.2, tangent loss = 0.0009) with a thickness of 0.254 mm had been utilized as a dielectric material with a copper cladding of 0.035 mm. The dimensions of the proposed unit cells are X = 3.3 mm, Y = 3.2 mm, X1 = 2.8 mm, Y1 = 2.7 mm, g = 0.35 mm, and W = 0.2 mm. To achieve the extraordinary characteristics of MM, the structure should be less than the wavelength of operation [27,28], which makes fabrication very challenging at the MMW spectrum.

CST Microwave Studio was used to simulate the proposed structure, where four electric walls of the waveguide were modeled as boundary conditions. The y-direction was used to propagate the EM wave with the electric field in the x-direction and magnetic field in the z-direction.

### 2.1. Simulation Results and Experimental Validation

**Figure 2:**
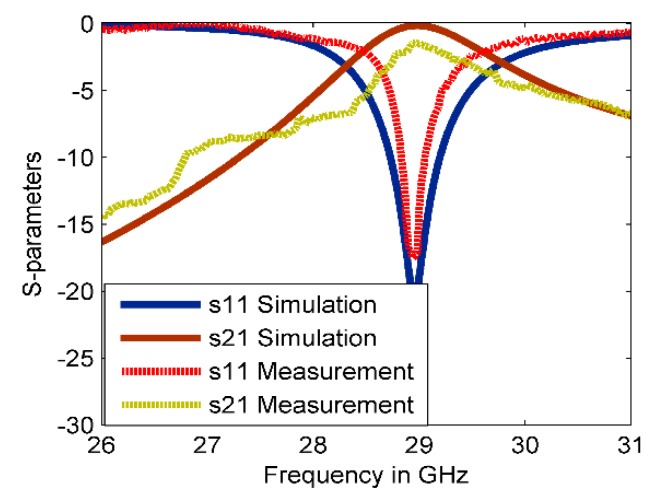
Reflection and transmission coefficients of the CSR structure.

The reflection and transmission coefficients of the proposed MM structure are plotted in Figure 2. It can be seen that the simulated reflection coefficient and bandwidth at 28.95 GHz are −20.45 dB and 0.5 GHz, respectively. The inherent loss is a serious issue in the MM structures which limits the range of their practical applications, especially at MMW frequency range. In this work, the transmission coefficient was used to measure the MM loss. Figure 2 shows that the loss in the simulated result was relatively small with −0.2 dB because of the proper geometrical arrangement of the structure. To validate the simulated results, the proposed MM structure is fabricated and measured as shown in Figure 2. Due to the small size of the fabricated sample at the high-frequency range, the waveguide measurement setup was used in this work. The WR-28 waveguide with two square-shaped covers was adopted as the transmitter and receiver ports. The proposed structure was suited precisely within the waveguide flanges. Thus, the transmission and the reflection coefficients could be obtained using this test setup. The experimental result of the reflection coefficient agrees well with the simulated result with reducing the resonance and bandwidth to −18 dB and 0.27 GHz, respectively. On the other hand, the measured result of the transmission coefficient has fluctuated over the whole range and deviates from the simulated results because of human error through the assembly of the MM periodic structure, the sensitivity of the measurement at high-frequency range, and the leakage of an EM wave between the two flanges of the waveguide. However, the measured result still agrees, to some extent, with the simulated result, especially for frequencies above 28 GHz.

### 2.2. Reconfigurable MM Structure

**Figure 3:**
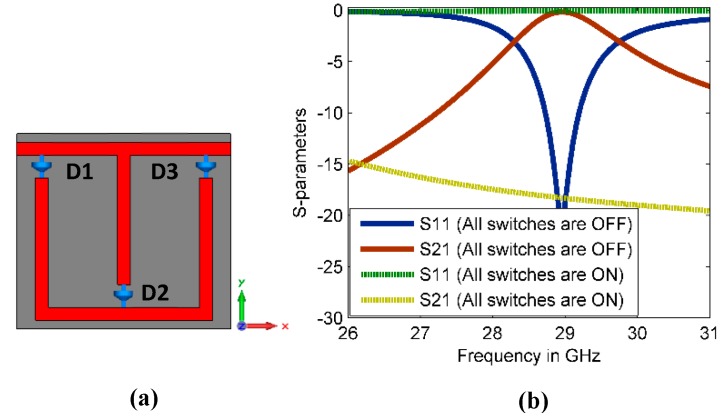
(**a**) Reconfigurable MM structure and (**b**) S-parameter results of the reconfigurable CSR structure.

The reconfigurable property of the proposed MM structure was achieved using three copper strips to mimic the dimensions of PIN diodes. Figure 3a shows the reconfigurable MM structure using three ideal switches (D1, D2, and D3), which are formed in the hiatuses of the three vertical bars of the structure. In the simulation, the copper material with the dimensions of (0.35 mm × 0.2 mm) is used to mimic the dimensions of the MA4AGFCP910 PIN diode. In this method, the ON state of the switch is represented by the copper strip, whereas vacuum represents the OFF state. The reflection and transmission coefficients of the reconfigurable CSR are depicted in Figure 3b. Only two studied cases met the requirements of this study. In the first case, when all switches are OFF, the resonance characteristics of OFF MM configuration are as the CSR without the reconfigurable property, which is discussed in Section 2.1. In the second case, when all switches are ON state, the resonance characteristics of ON MM configuration differ as illustrated in Figure 3b. To retrieve the refractive index of the reconfigurable CSR, the robust retrieval method has been used [29].

**Figure 4:**
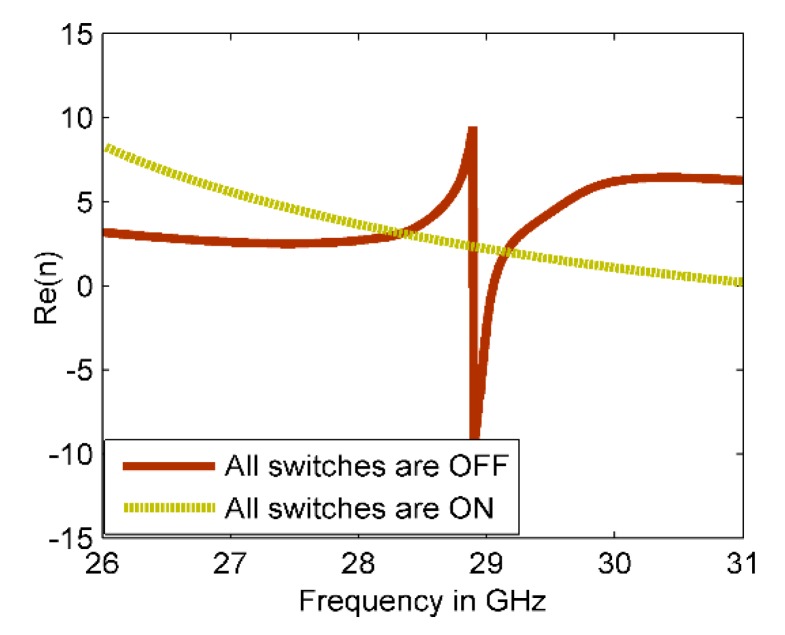
The real index of refraction of the reconfigurable CSR structure.

The real refractive indices of the reconfigurable structure for both MM configurations, ON and OFF, are shown in Figure 4. It can be seen that the retrieved refractive indices differ at 28 GHz. For OFF MM configuration, the refractive index is about 2.7, whereas the refractive index of ON MM configuration changes to 3.6.

The reconfigurable MM using ideal switches is used as proof of concept. It should be pointed out that the execution of practical reconfigurability using real PIN diode is way beyond the scope of this work due to the lack of equipment for achieving such a process, and we will just present the potential of the structure to achieve reconfigurability.

## 3. MM for Beam Deflection Antenna

### 3.1. Dipole Antenna Design

**Figure 5:**
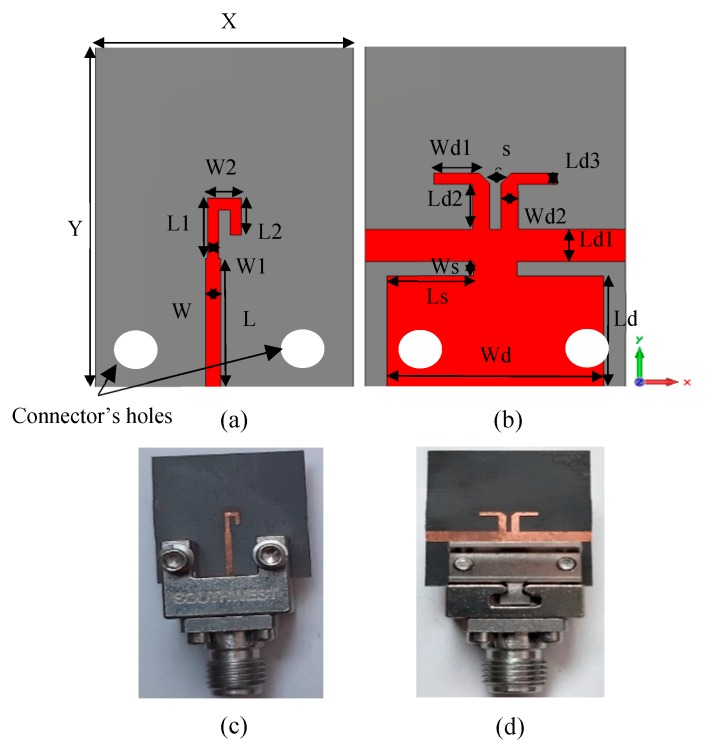
The proposed dipole (**a**,**b**) the front and back views of the designed configuration and (**c**,**d**) the front and back views of the fabricated prototype.

**Figure 6:**
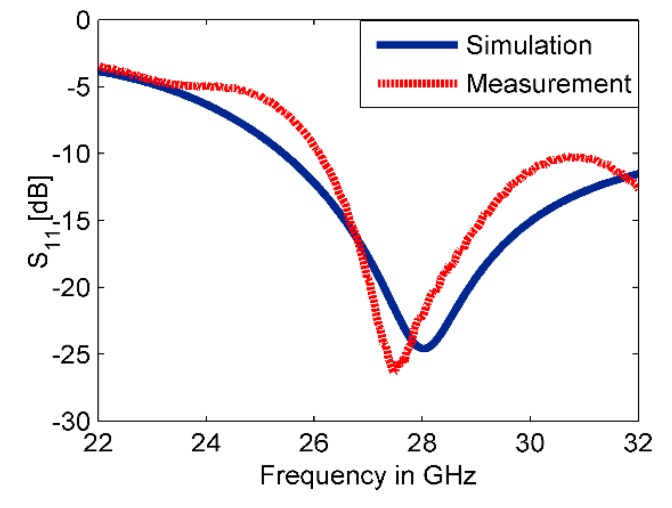
The reflection coefficient of the dipole antenna.

**Figure 7:**
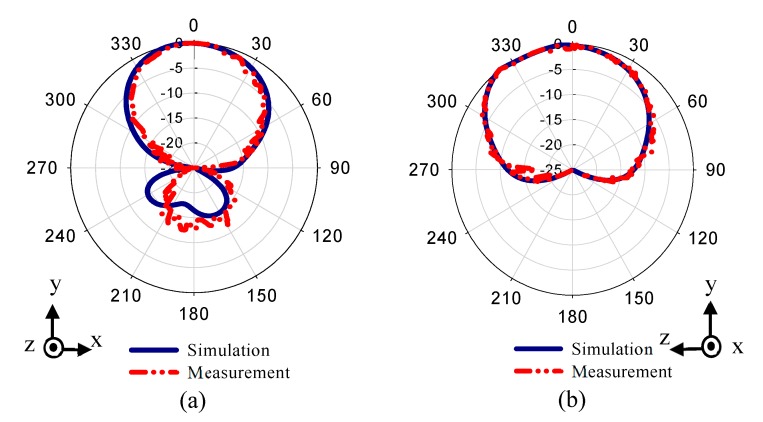
Normalized radiation patterns of the dipole antenna at 28 GHz: (**a**) E-plane (xy) and (**b**) H-plane (zy).

This work presents a method for tilting an antenna beam in positive and negative directions using reconfigurable MM at 28 GHz. Figure 5a,b present schematic views of the updated printed dipole antenna version [30]. The feeding line is printed on the front side of the dielectric layer as shown in Figure 5a. The dipole arms are printed and separated by slot s at the backside of the substrate as displayed in Figure 5b. The dielectric layer is Rogers RT5880 with relative permittivity 2.2, tan δ of 0.0009, and a thickness of 0.254 mm. The strip with dimensions of X = 12 mm and Ld1 = 5.3 mm, that extends along the x-axis under the two dipole arms, helps to provide a directional radiation pattern in E-plane. This strip acts as a reflector for guiding the EM wave toward the end-fire direction of the antenna (y-axis). This reflector should be longer than the two dipole arms for reflecting the radiation to the end-fire direction. Thus, a directional radiation pattern in E-plane can be obtained. The directional antenna is preferred for beam switching capability [31]. The xy-plane is the azimuth plane (E-plane), while the zy-plane represents the elevation plane (H-plane) [22]. The geometric specifications of the proposed antenna are described in Table 1. The overall size of the antenna is relatively small with dimensions of 1.1$\;\lambda_{0}\;$× 1.49$\;\lambda_{0}$. The prototype of the antenna was fabricated as shown in Figure 5c,d and measured to verify the proposed design. Figure 6 shows the simulated and measured reflection coefficients. The proposed antenna operates at 28 GHz with $S_{11}\;$of −24.2 dB and wide bandwidth. There is a good match between the simulated and measured results. However, a small downshift in the measured results was observed due to the fabrication tolerance, the sensitivity of measurement at the high-frequency range, and the effect of the end-launch connector that exhibits measurement error. Figure 7 displays that the E-plane radiation pattern of the dipole is directional at 28 GHz. Also, the antenna achieved a peak gain of 5.12 dB at 28 GHz. The simulation and measurement results show good agreement for the E-plane (xy) and H-plane (zy) at 28 GHz.

### 3.2. Theoretical Basis of Beam Deflection

**Figure 8:**
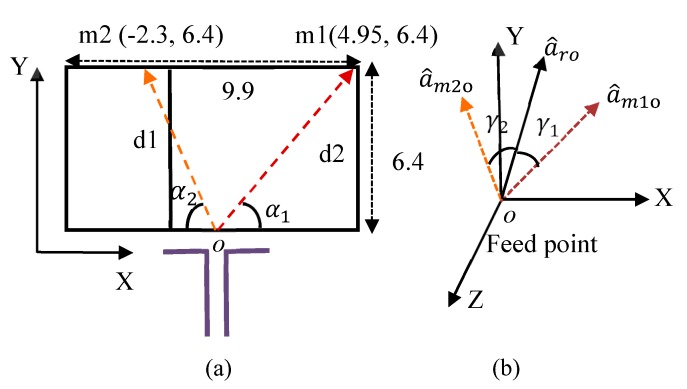
The EM ray routes and their locations from the feed point for array factor and radiation pattern calculations (**a**) on the structure and (**b**) on the coordinate plane.

The theoretical concept of radiation beam deflection relies on two MM configurations of different refractive indices, which were placed in the way of the EM rays. According to Snell’s law, when the EM wave travels into two mediums of different refractive indices, it refracts at a predefined angle. The various refractive indices of the reconfigurable MM create phase change for the EM wave, which leads to beam deflection. This concept is used here to tilt the radiation beam of the dipole antenna in E-plane (xy). The best way to obtain mediums with different refractive indices on the finite area of the substrate is by using suitable MM design with reconfigurability property. The two configurations of different refractive indices are positioned in the proximity of EM source (dipole antenna) as shown in Figure 8. The 2 × 3 unit cells of reconfigurable MM are inserted in the front of the dipole antenna with overall dimensions of 9.9 mm × 6.4 mm. The rays of an EM wave travel over the reconfigurable MM structure with different lengths and directions. As described in [32], the calculation of the array factor (AF) at far-field is used to determine the resultant effect of each ray. The position of each element is depicted in Figure 8.

The AF can be expressed as

(1) $$\mathrm{AF}=1+e^{ikd_{1}\cos\gamma_{1}}+e^{ikd_{2}\cos\gamma_{2}}$$

where k and d are the wavenumber and the length of each ray, respectively. The ray vectors ${\hat{a}}_{m1o}$ and ${\hat{a}}_{m2o}\;$that extends from the feed point *o* to the two MM configurations are given by

(2) $${\hat{a}}_{m1o}=0.61{\hat{a}}_{x}+0.78{\hat{a}}_{y}$$

(3) $${\hat{\;a}}_{m2o}=-0.33{\hat{a}}_{x}+0.94{\hat{a}}_{y}$$

where $\alpha_{1}$= 52.2° and $\alpha_{2}$= 70.2°.

The unit vector of the coordinate plane is given by

(4) $${\hat{a}}_{ro}=\;sin\theta cos\varphi {\hat{a}}_{x}+sin\theta sin\varphi {\hat{a}}_{y}+cos\theta {\hat{a}}_{z}$$

The angles $\gamma_{1}$ and $\gamma_{2}$ in Figure 8b are created between the two ray vectors ${\hat{a}}_{m1o}$ and ${\hat{a}}_{m2o}$ and unit vector ${\hat{a}}_{ro}$ and obtained by the dot product as follows:

(5) $$\cos\gamma_{1}={\hat{a}}_{m1o}.{\hat{a}}_{ro}=\;0.61sin\theta cos\varphi +0.79sin\theta sin\varphi$$

(6) $$\cos\gamma_{2}={\hat{a}}_{m2o}.{\hat{a}}_{ro}=\;-0.33sin\theta cos\varphi +0.94sin\theta sin\varphi$$

At θ = 90° plane, Equations (5) and (6) are reduced to

(7) $$\;\cos\gamma_{1}=\;0.61cos\varphi +0.79sin\varphi$$

(8) $$\cos\gamma_{2}=\;-0.33cos\varphi +0.94sin\varphi$$

(9) $$\mathrm{AF}=1+e^{ikd_{1}\left(0.61cos\varphi +0.79sin\varphi \right)}+e^{ikd_{2}\left(-0.33cos\varphi +0.94sin\varphi \right)}$$

**Figure 9:**
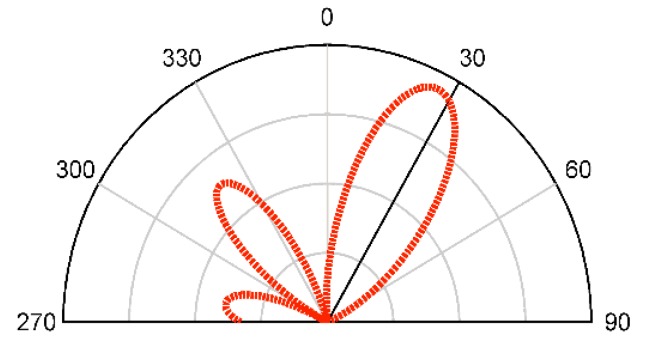
The radiation pattern of the proposed antenna loaded by reconfigurable MM.

The radiation pattern of the dipole antenna loaded by reconfigurable MM structure is calculated by multiplying the AF and the dipole antenna element factor which is given by cos((π/2)cosφ)/sinφ [32]. The dipole antenna’s main beam is deflected at an angle of 28° when the reconfigurable MM unit cells are loaded as depicted in Figure 9.

### 3.3. Antenna Beam Deflection

In this work, the idea behind using the MM for beam deflection antenna is that the two configurations of MM are placed in the same substrate of antenna next to the radiating element to provide different refractive index values (ON and OFF with refractive indices of 3.6 and 2.7, respectively). When the EM wave passes through ON and OFF MM unit cells, it faces different refractive index values, thereby producing different phases which, in turn, leads to deflection of the beam toward the high refractive index (ON MM configuration). 

The reconfigurable MM unit cells are placed in the xy-plane of the dipole antenna. The antenna acts as the source of the EM wave that passes thought the reconfigurable MM along the y-direction, which is the propagating mode of the MM structure as explained in Section 2. In other words, the reconfigurable MM unit cells are placed in the E-plane (xy). From the MM design in Section 2, the two ports that used to propagate the EM wave through the CSR structure are assigned in the y-direction. It is the same direction of the EM wave that emits from the dipole antenna. Thus, the EM wave is deflected by the MM array in the E-plane only (not in the H-plane). To perform beam deflection angles in both directions, 2 × 3 unit cells of reconfigurable MM are inserted in the same substrate of the printed dipole antenna with different arrangements. 

**Figure 10:**
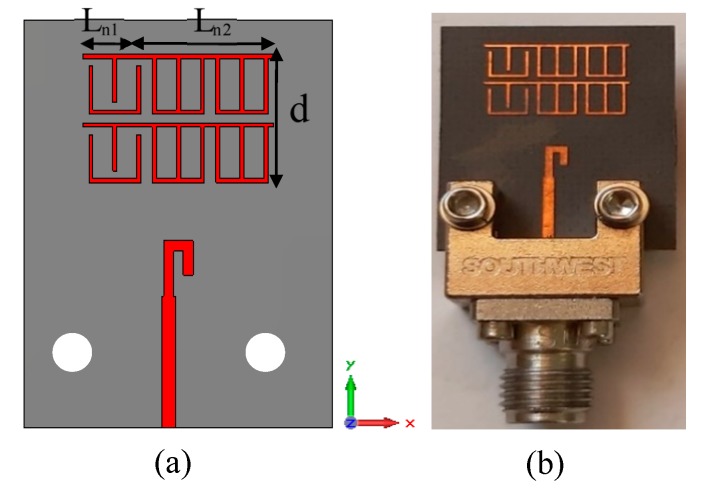
Integration of 2 × 3 MM array with the proposed antenna for positive deflection (**a**) schematic view of MM antenna and (**b**) photo of the fabricated prototype.

**Figure 11:**
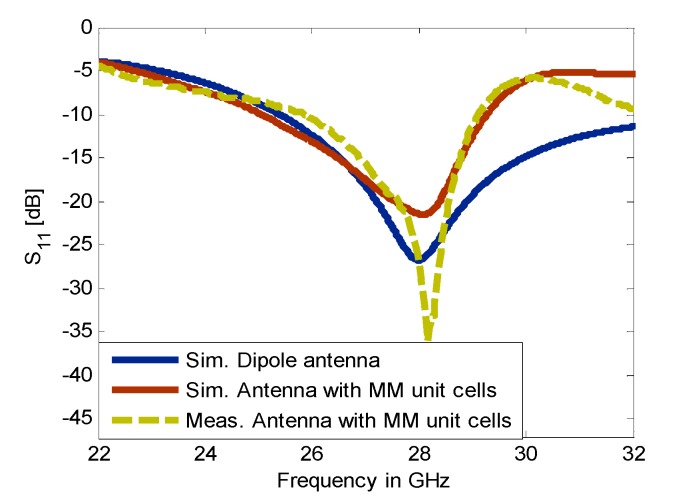
The simulated and measured reflection coefficient of the dipole antenna and MM antenna during the positive deflection.

The configuration of the dipole antenna with 2 × 3 MM array for positive deflection in E-plane and a photo of the fabricated prototype are depicted in Figure 10a,b, respectively. The 2 × 2 unit cells with ON configuration extend from the center to the right side of the substrate at a length of $L_{n2\;}\;$= 6.6 mm, while the 2 × 1 unit cells with OFF configuration were placed to the left side of the substrate at a length of $L_{n1}\;$= 3.3 mm. This arrangement led to two refractive index configurations in the vicinity of the radiating elements and thereby deflected the main beam toward the MM configuration with a high refractive index [26]. The distance between the antenna feeding and the MM array is optimized to be 2.7 mm. The reflection coefficients of the dipole antenna and MM antenna have been plotted in Figure 11. It is noticeable that inclusion of the MM configurations influences the reflection coefficient of the antenna in comparison with that of the dipole antenna; nevertheless, the reflection coefficient of the antenna loaded by MM unit cells is kept at less than −10 dB at 28 GHz. To verify the simulated results, the MM antenna was fabricated and tested.

The southwest end-launch connector of 1.85 mm had been used in the measurements to verify the antenna characteristics. The measured reflection coefficient shows good consistency with the simulated results, with an increase in the $S_{11}\;$up to −37 dB.

**Figure 12:**
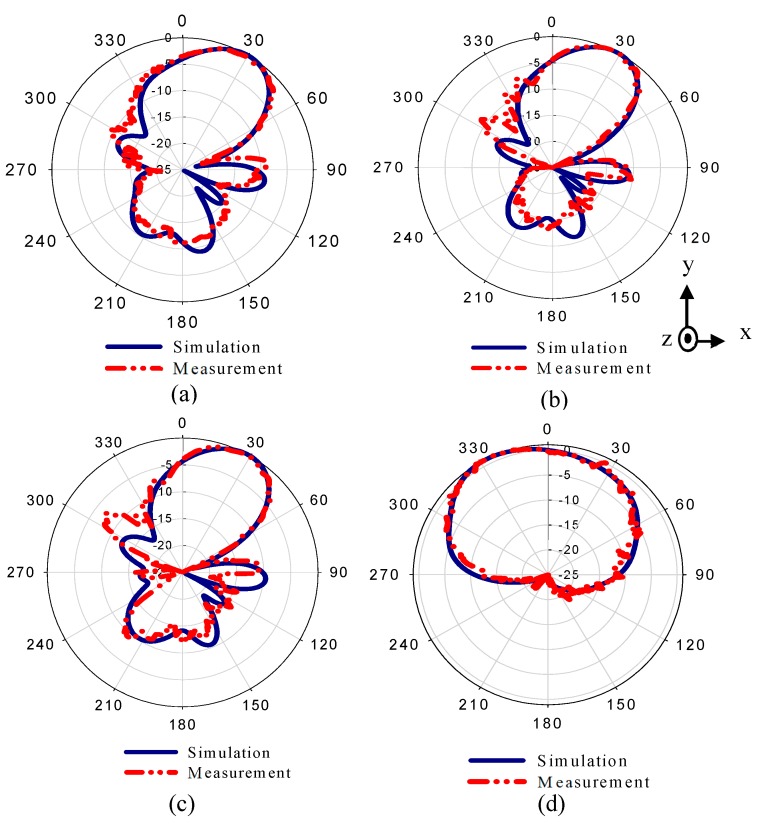
Normalized simulated and measured radiation patterns of the MM antenna in E-plane (xy) at (**a**) 27.7 GHz, (**b**) 28 GHz, and (**c**) 28.3 GHz, and (**d**) in H-plane at 28 GHz.

The radiation patterns of the dipole antenna with two MM configurations are displayed in Figure 12. The rays that were emitted from the source of the EM wave—the dipole antenna—passed through the different refractive index configurations, resulting in the deflection of the main beam toward a high refractive index configuration (+y). Figure 12a–d show the radiation patterns of the MM antenna in E-plane at 27.7, 28, and 28.3 GHz, and in H-plane at 28 GHz.

**Figure 13:**
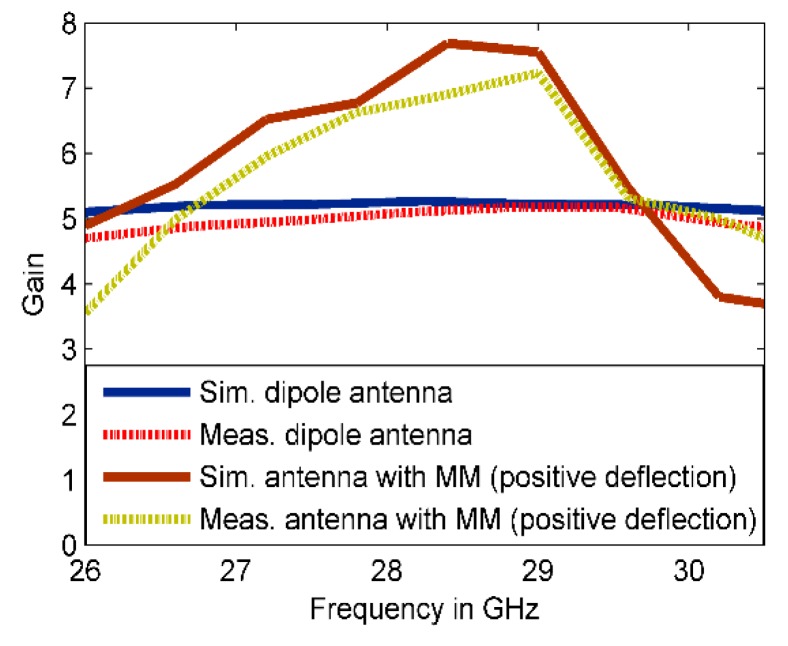
Simulated and measured gain of the antenna and MM antenna for positive deflection at 28 GHz.

**Figure 14:**
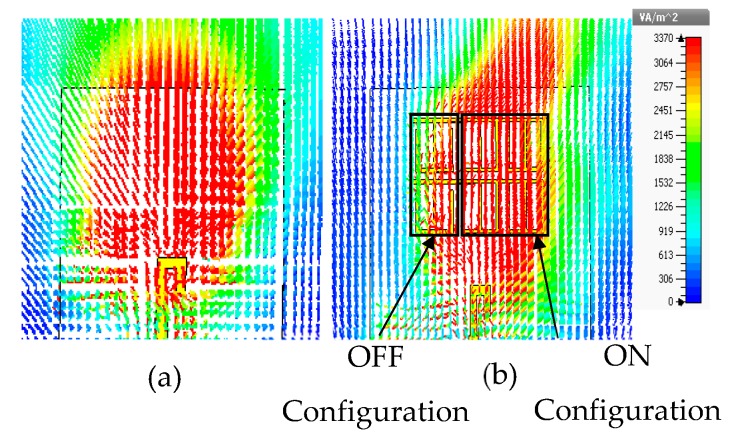
Radiation power flow in E-plane at 28 GHz: (**a**) dipole antenna; (**b**) MM antenna.

The simulated results show that the main beam is deflected by an angle of +30°. The measured radiation pattern in E-plane confirms that the direction of the beam is deflected by +30°. Furthermore, this deflection is accompanied by gain enhancement of 1.9 dB as shown in Figure 13. Figure 13 depicts simulated and measured gain of the dipole antenna and MM antenna for positive deflection at 28 GHz. The gain improvement is very clear for MM antenna compared to that of the dipole antenna. The discrepancy between the measured and simulated results is due to the fabrication tolerance and measurement error. The normalized radiation patterns of the antenna and MM antenna in H-plane at 28 GHz is displayed in Figure 12d. As expected, no clear deflection is observed. To better understand the beam deflection mechanism, the distribution of radiation power flow (the Poynting vector) over the dipole antenna and MM antenna in the E-plane was simulated and plotted in Figure 14. The power flow of the dipole antenna without the MM array reveals that the antenna beam was radiated with no deflection. By contrast, the distribution of power flow reveals that when the different MM configurations were inserted into the antenna substrate, the radiation beam of the antenna was deflected toward the ON MM configuration.

**Figure 15:**
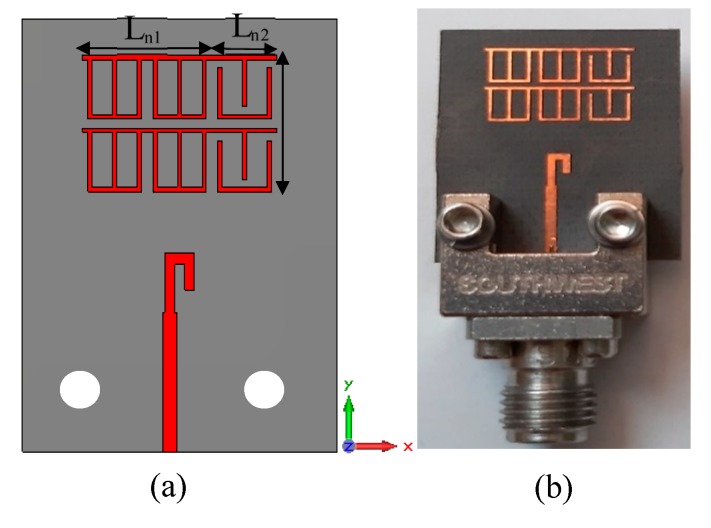
Integration of 2 × 3 MM array with the proposed antenna for negative deflection: (**a**) designed configuration and (**b**) fabricated sample.

**Figure 16:**
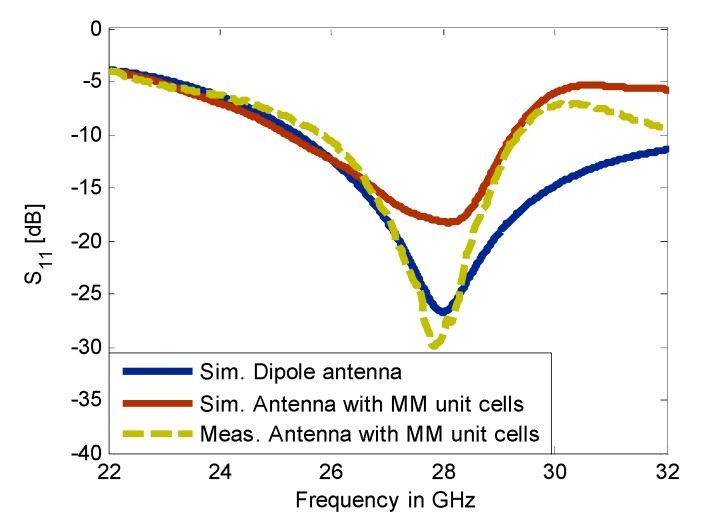
The simulated and measured reflection coefficient of the dipole antenna and MM antenna during the negative deflection.

To carry out a negative deflection angle, the arrangement of the MM configurations is reversed compared to that of the positive deflection angle. Figure 15a depicts the proposed antenna incorporated with 2 × 3 MM unit cells for negative deflection in E-plane. The 2 × 2 unit cells with ON configuration spread from the center to the left side of the substrate at a length of $L_{n1}\;$= 6.6 mm and the 2 × 1 unit cells with OFF configuration were placed to the right side of the antenna at a length of $L_{n2}\;$= 3.3 mm. Figure 16 reveals the dipole antenna and MM antenna performances in terms of reflection. Good matching is shown between the reflection coefficients of a dipole antenna and MM antenna whereas the $S_{11}\;$remains below −10 at 28 GHz. However, the embedding of MM array onto the antenna substrate causes an obvious deviation, especially above 28 GHz.

**Figure 17:**
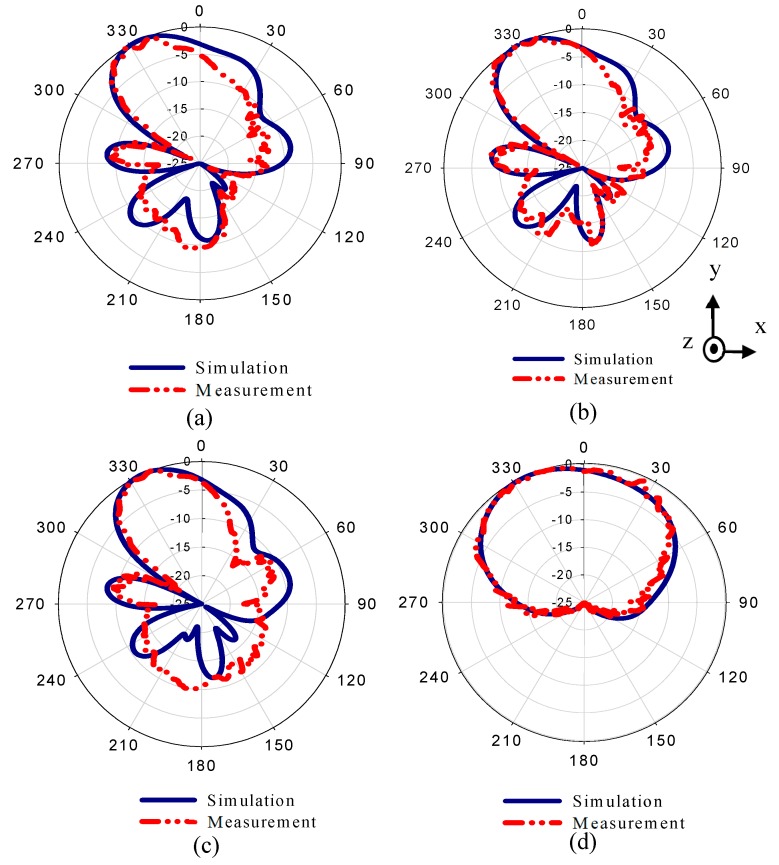
Normalized numerical and experimental radiation patterns of the dipole antenna embedded by two configurations of MM in E-plane (xy) at (**a**) 27.7 GHz, (**b**) 28 GHz, and (**c**) 28.3 GHz, and (**d**) in H-plane at 28 GHz.

**Figure 18:**
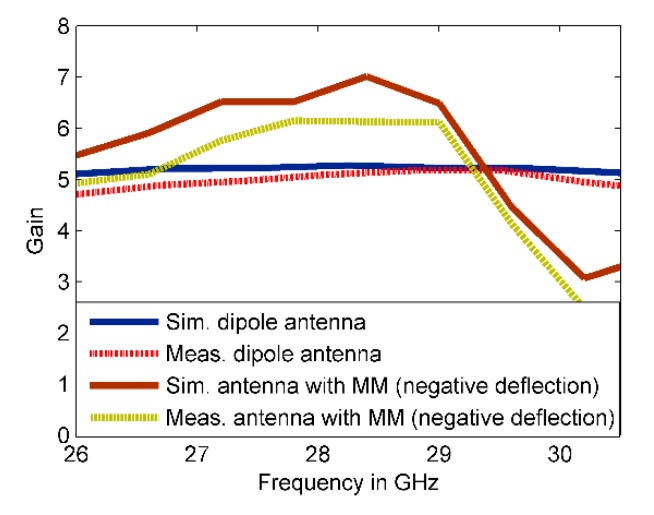
The numerical and experimental gain of the proposed antenna with and without MM array for negative deflection angle at 28 GHz.

**Figure 19:**
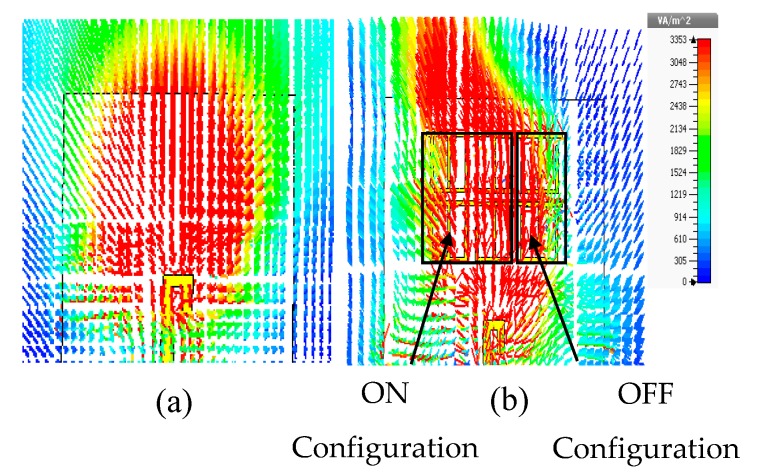
Radiation power flow in E-plane at 28 GHz: (**a**) dipole antenna cells; (**b**) MM antenna.

The measured reflection coefficient agrees well with the simulated one, with an increase in the reflection coefficient up to −35 dB. On the other hand, the radiation patterns of the proposed MM antenna in E-plane are plotted in Figure 17a–c at 27.7, 28, and 28.3 GHz, respectively. The radiation pattern in H-plane at 28 GHz is shown in Figure 17d. The simulated result shows that the main beam is deflected by an angle of −27° at 28 GHz. Both numerical and experimental results show good agreement at the 27.7, 28, and 28.3 GHz. However, a small deviation in the measured results was observed due to fabrication tolerance. The 3° difference between the positive and negative deflection angles is due to the off-center feed point of the proposed antenna. Through the negative defection, there is a gain enhancement by 1.5 dB. The numerical and experimental gain of the antenna and MM antenna for negative deflection at 28 GHz is illustrated in Figure 18. The fabrication tolerance and the measurement errors induced by measurement equipment affect the measured gain and cause a discrepancy in measured results compared to that of simulated results. To explain the negative deflection in terms of power flow, Figure 19a, b depict the power flow distribution at 28 GHz for both the dipole antenna and MM antenna. Figure 19b displays that the deflection in the main beam is toward the ON MM configuration.

Table 2 presents the comparison of the recent literature with this work in terms of antenna, frequency of operation, radiation pattern tilt angle, gain enhancement, and the MM shape used. The proposed antenna with MM array produces high deflection angles with an acceptable increase in the gain in both directions compared to other reported literature.

## 4. Conclusions

A reconfigurable CSR MM structure was proposed to operate in the MMW spectrum. The CSR was reconfigured to provide two configurations with different refractive indices. These configurations cooperated with the dipole antenna to tilt the radiation beam in the E-plane. The dipole antenna was optimized to operate at a 28 GHz band with wide bandwidth. A 2 × 3 array of reconfigurable MM was inserted on the antenna’s dielectric layer to perform positive and negative deflection angles. A dipole antenna with an MM array for both positive and negative deflection angles is fabricated and measured. The measured results of the radiation patterns demonstrate that the main beam was deflected by angles of +30° and −27° in the E-plane depending on the arrangement of the two MM configurations on the antenna substrate. Furthermore, the gain increased by 26.7% and 22.4% for both positive and negative deflection angles, respectively. The reflection coefficients were better than −10 dB for all deflection angles. The proposed structure is a promising candidate for beamforming applications at the 5G candidate band of 28 GHz.

## Figures and Tables

**Table 1:** Geometric specifications of the printed dipole antenna.

Parameter	Value (mm)	Parameter	Value (mm)
X	12	Ld	5.3
Y	16	Ws	0.7
L	5	Ls	4
L1	2.6	Ld1	1.5
L2	1.6	Ld2	2.1
W	0.7	Ld3	0.5
W1	0.5	Wd1	2.1
W2	1.55	Wd2	0.8
Wd	10	s	0.5

**Table 2:** Comparison of the present work with that reported literature for beam deflection using MM structures. SSR = split-ring resonator.

Ref.	Antenna Type	Frequency Band	Deflection Angle (degrees)		MM Unit Cell
			Gain (dB)		
			Positive Def.	Negative Def.	
			Positive Gain	Negative Gain	
[17]	Patch antenna	C-band (7.3 GHz)	15	-	SRR
			Reduced by 1.5	-	
[18]	Bow-tie antenna	C-band (7.5 GHz)	17	-	H-shape
			Enhanced by 2.7	-	
[19]	Horn antenna	Ku- band (15 GHz)	10	-	SRR and H-shape
			Reduced by 0.48	-	
[20]	Dipole antenna	V-band (60 GHz)	30	-	HRIM
			Enhanced by 5	-	
[21]	Leaky-wave antenna	X-band8 GHz	15	15	J-shaped MM
			-	-	
[22]	Dipole antenna	S-band3.5 GHz	25	24	ASSR
			Enhanced by 3	Enhanced by 2.7	
This work	Printed dipole antenna	Ka-band (28 GHz)	30	27	CSR
			Enhanced by 1.9	Enhanced by 1.5	
